# Too Fast for Spin Flipping: Absence of Chirality-Induced Spin Selectivity in Coherent Electron Transport through Single-Molecule Junctions

**DOI:** 10.1021/jacs.5c08517

**Published:** 2025-07-02

**Authors:** Liang Li, Wanzhuo Shi, Ankit Mahajan, Junxiang Zhang, Marta Gómez-Gómez, Jorge Labella, Shayan Louie, Tomás Torres, Stephen Barlow, Seth R. Marder, David R. Reichman, Latha Venkataraman

**Affiliations:** † Department of Chemistry, 5798 Columbia University, New York, New York 10027, United States; ‡ Renewable and Sustainable Energy Institute (RASEI), 1878 University of Colorado Boulder, Boulder, Colorado 80309, United States; § 16722 Department of Organic Chemistry. Universidad Auto’noma de Madrid, Campus de Cantoblanco, C/Francisco Toma’s y Valiente 7, Madrid 28049, Spain; ∥ Institute for Advanced Research in Chemical Sciences (IAdChem), Universidad Auto’noma de Madrid, Madrid 28049, Spain; ⊥ IMDEA-Nanociencia, Campus de Cantoblanco, Madrid 28049, Spain; # Departments of Chemical and Biological Engineering and of Chemistry, University of Colorado Boulder, Boulder, Colorado 80309, United States; ¶ Department of Applied Physics and Applied Mathematics, Columbia University, New York, New York 10027, United States; ∇ 148492 Institute of Science and Technology Austria, Klosterneuburg 3400, Austria

## Abstract

Chirality-induced spin selectivity (CISS), which refers to the ability of chiral molecules to preferentially select spins during electron transfer, has attracted great attention during the past two decades. However, the theoretical and experimental understanding of the CISS effect remains preliminary. In this study, we demonstrate that there is no distinguishable CISS effect in the case of coherent electron transport through single chiral molecular junctions for a set of four molecule studied here. Our conclusion is based on statistical evaluations of thousands of single-molecule junctions across four different molecules with different origins of chirality measured by the scanning tunneling microscope-based break-junction technique. The experimental results for all molecules show no dependence on external magnetic field or chirality in both conductance and current–voltage measurements. In addition, *ab initio* Hartree-Fork calculations combined with the nonequilibrium Green’s function method reveal that the spin–orbit coupling within chiral junctions bound to a few gold atoms is generally too weak to induce detectable spin polarizations from spin flipping or spin filtering during the ultrafast electron-transport time scale. The absence of an observable CISS effect in the coherent electron-transport regime suggests that the effect may only be found in other electron-transfer regimes and requires further experimental and theoretical efforts to achieve a comprehensive understanding.

## Graphical abstract

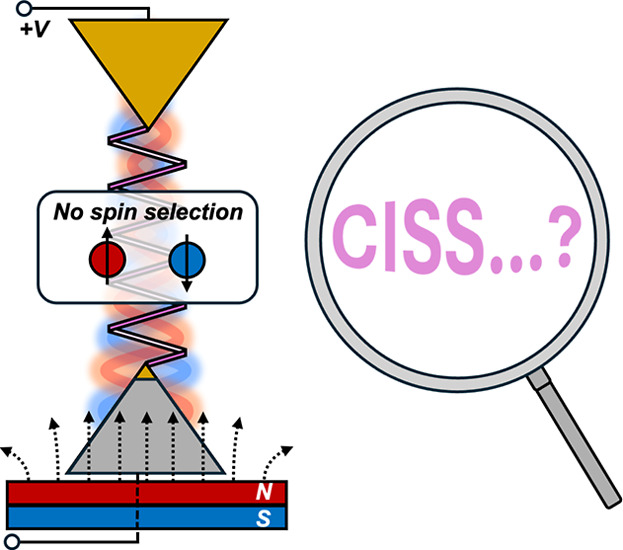

## Introduction

Chirality-induced spin selectivity (CISS) refers to a phenomenon wherein the chirality, or handedness, of molecular structures preferentially selects electrons with a specific spin polarization during a range of electron-transfer mechanisms.^1–6^ This interesting effect connects the structural asymmetry of molecules to the spin asymmetry in electron transport. CISS was first observed through spin-polarized photoemission resulting from asymmetric scattering within thin films of chiral molecules deposited on gold.^7^ Since then, extensive explorations of the CISS effect have been conducted using different chiral crystals,^8–11^ polymers^12,13^ and chiral molecules,^14–16^ including chiral biomolecules such as nucleic acids^17–20^ or peptides.^21–23^ Despite significant experimental advancements over the past two decades, the theoretical understanding of CISS remains preliminary.^5^ It has been proposed that spin scattering within chiral molecules is induced by spin–orbit coupling (SOC).^24,25^ However, the SOC effect in organic molecules containing only light atoms is too weak^26^ to account for the significant spin selectivity observed from experiments. Recently, Fay and Limmer established theoretical models for the photoinduced CISS effect in chiral donor–acceptor molecular systems.^27,28^ However, their models are limited to the incoherent electron-hopping regime and do not adequately explain the CISS effect in coherent electron transport reported in break junction^17,29^ or conductive atomic-force microscopy (AFM) measurements of monolayers on a magnetized substrate.^22,30–33^

In coherent transport through single-molecule junctions, the total electron transmission, *T*, is the sum of spin-up and spin-down transmission probabilities, *T* = *T*_↑_+*T*_↓_. For closed-shell molecules with degenerate spin orbitals, each spin channel contributes equally at 50% to the total electron transmission. Therefore, even if a chiral molecule acts as a 100% spin filter due to the CISS effect (by selectively scattering one spin), it can only block up to 50% of the total electron transmission. If the chiral molecule instead functions as a spin torque, the resulting difference in total electron transmission would be even smaller. In molecular junctions, this modest change in conductance or current due to spin selection is often overshadowed by the junction geometric fluctuations. For measurements of thousands of different molecular junctions of the same molecular species, although the most probable conductance (distribution peak) can be defined accurately, the statistical broadening of the distribution often approaches or exceeds 1 order of magnitude.^34–38^ Indeed, because of the nonlinear current–voltage characteristics of single molecules,^39,40^ small perturbations to molecular-junction structure or environment can lead to significant changes in conductance. If the number of measured molecular junctions is insufficient to provide a statistically significant data set, the results may yield misleading conclusions regarding the CISS effect in chiral molecules, particularly given the subtlety of this effect.

In this study, we demonstrate that there is no distinguishable CISS effect in coherent electron transport through single chiral molecular junctions using the scanning tunneling microscope-based break-junction (STM-BJ) technique at room temperature.^41,42^ We arrive at this seemingly controversial conclusion through careful evaluations of statistical data sets over thousands of single-molecule junctions, where we show that the chirality of the molecular backbone does not alter the junction conductance or current–voltage characteristics sufficiently to distinguish from the measured distributions. The chiral molecules tested in this work encompass a variety of molecular backbones including aromatic and aliphatic structures, chiral bridges and linker attachments, stereogenic centers and axes, including chirality arising from the inherent curvature of the molecular structure, and include molecules from both commercial and laboratory sources. This diverse selection ensures that the absence of observable CISS effects is not limited to a specific type of chiral molecule or synthetic route but is a general phenomenon across different molecular configurations and structural features. We then performed *ab initio* calculations of the chiral molecules including SOC, and incorporated them with the nonequilibrium Green’s function method to investigate the effect of SOC in spin-dependent transmission functions. These calculations show negligible spin polarizations in transmissions at the Fermi energy, in good agreement with our experimental results. Our findings suggest that the CISS effect may be more complex and regime-dependent than previously thought, requiring further investigation of its mechanisms and limitations.

## Experimental Section

We use the STM-BJ technique to measure current and conductance through single-molecule junctions.^41,42^ In the context of CISS, molecules with opposite chiralities are expected to exhibit opposite spin selectivity, i.e. if the right-handed molecule selectively transfers spin-up electrons, its left-handed counterpart should exhibit the same extent of selectivity for spin-down electrons.^1^ In the STM-BJ technique, a Au tip and a Au-coated substrate (mica or steel puck) are used as electrodes to form single-molecule junctions (Figure 1A). However, since Au is diamagnetic, the electric current driven through the molecular junctions is not spin-polarized. Therefore, with a nonmagnetic STM-BJ setup (Figure 1A), regardless of whether the CISS effect exists or not, molecules with opposite chiralities should always have the same measured molecular conductance under identical measurement conditions. To probe the ability of chiral molecules to act as spin filters, we therefore used a magnetic STM-BJ setup (Figure 1B), in which the Au-substrate was replaced by a magnetic heterostructure consisting of Ti­(10 nm)/Ni­(100 nm)/Au­(8 nm) on Si. During the measurements, the magnetic substrate was fixed onto a NdFeB permanent magnet, which magnetizes the Ni­(100 nm) layer to a preferred magnetization orientation, generating a homogeneous magnetic field (either +B or −B) of at least 0.3 T perpendicular to the substrate as measured by a magnetometer. The thin Au layer (8 nm) on top of Ni increases the feasibility of forming single-molecule junctions without significantly affecting the extent of spin polarized current through this layer, as the spin-coherence length in Au is approximately 30 nm at room temperature.^43^ Note that this heterostructured magnetic substrate has been commonly used in many published works from which the CISS effect was observed.^23,30,32,44–46^

**Figure 1:**
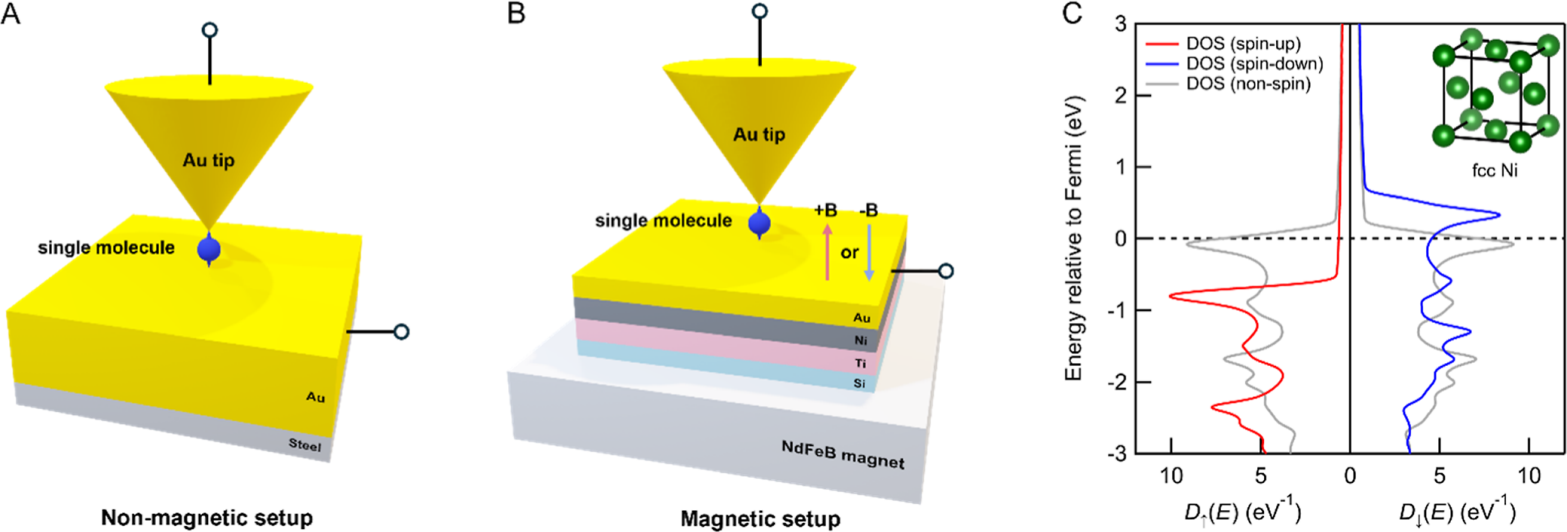
Schematic of (A) nonmagnetic STM-BJ setup and (B) magnetic STM-BJ setup. (C) DFT calculated Kohn–Sham density of states (KS DOS) of face-centered cubic Ni with upward magnetization. The spin-up DOS is indicated red, and the spin-down DOS is indicated blue. The gray dashed line indicates the KS DOS of demagnetized Ni.

Because Ni is a transition metal with unpaired d-electrons, its spin-up and spin-down sub-bands have different densities of states at the Fermi level upon magnetization, enabling it to inject spin-polarized current into molecular junctions. This is clearly visible in the density functional theory (DFT)-based density of states shown in Figure 1C calculated using FHI-Aims DFT code for face-centered cubic Ni crystal with the PBE functional.^47–50^ With opposing magnetization in Ni, the populations of spin-up and spin-down electrons are reversed. For a given chirality of a molecule in the junction that selectively filters one spin channel, the molecular conductance measured with the magnetic setup should depend on the magnetic field orientations (+B and −B). To ensure that the observed differences in conductance are solely due to the CISS effect, it is also crucial to conduct the same experiment with the opposite enantiomer to confirm whether the conductance results are reversed under +B and −B fields. In general, the CISS effect requires the molecular conductance, *G*, to follow an inversion relation, *G*(*R*,+B) = *G*(*S*,−B) and *G*(*R*,−B) = *G*(*S*,+B), where *R* and *S* denote the two chiral enantiomers. Furthermore, since Au has an equal mixture of 50% spin-up and 50% spin-down electrons, the molecular conductance measured with the nonmagnetic setup should in principle lie between the conductance values of the same molecule measured under + B and −B fields using the magnetic setup.

The spin polarization of Ni can be utilized to evaluate the maximum change in molecular conductance under different magnetic field orientations. Spin polarization (*P*) is defined as the ratio between spin current and electron current, *P*=(*I*_↑_−*I*_↓_)/(*I*_↑_+*I*_↓_), where *I*_↑_ and *I*_↓_ are defined as the spin-up current and the spin-down current, respectively. The spin polarization of Ni (*P*_Ni_) in tunnel junctions, which varies with different crystallographic directions and different surface morphologies of Ni,^51^ has been experimentally determined to range between 25% and 46%.^52–54^ Additionally, the density of states of Ni has a strong energy dependence near Fermi. This can influence the transmission of spin-polarized electrons across a single-molecule junction especially when a large bias voltage is used in the experiments. Based on these reasons, it is nontrivial to determine *P* for each junction. Instead, we assume that *P* in our magnetic setup falls between 25% and 46%, not accounting for any decoherence through the thin gold layer. We first consider a simplistic and extreme scenario where chiral molecules function as 100% spin filters. In this case, the maximum observable ratio in conductance between *R* and *S* enantiomers should range between 1.6 and 2.7, quite different from what has been reported in earlier works.^29,55^ However, in real experiments, since chiral molecules cannot be assumed to be perfect spin filters, the difference in conductance should be significantly lower than this estimated range.

## Results and Discussion

We first present results from measurements of (1*S*)- and (1*R*)-4,4′-bis­(4-(methylthio)­phenyl)-2,2′-dimethoxy-1,1′-binaphthalene molecules, labeled **1** ***S*** and **1** ***R***, Figure 2A, synthesized as described in Supporting Information Section I. These molecules feature oligomeric aromatic backbones, with chirality arising from the steric hindrance that restricts rotation of their central single bonds, which creates stereogenic axes and makes them atropisomers.^56,57^ This type of chiral structure has been reported to exhibit a CISS effect in donor-bridge-acceptor systems based on ensemble measurements.^16^ In our experiments, the two aurophilic–SMe groups added to the ends of the molecules serve to form molecular junctions with Au electrodes. We first measured the conductance of **1** ***S*** and **1** ***R*** in 1,2,4-trichlorobenzene (TCB) solution under +B and −B magnetic fields using the magnetic setup with 1 V applied tip bias. Both molecules showed a most probable conductance of 2.4 × 10^–6^ *G*_0_ (where *G*_0_ = 2 × 10^2^/*h* is a conductance quantum) and almost identical one-dimensional (1D) conductance histograms and two-dimensional (2D) conductance-displacement histograms (see Supporting Information Figure S3). This indicates that the electronic properties of **1** ***S*** and **1** ***R*** are the same at this bias voltage, regardless of the external magnetic-field orientations. The low conductance of these molecules is likely due to minimal π-conjugation caused by the sterically induced near-orthogonality of the naphthalene planes.^58^

**Figure 2:**
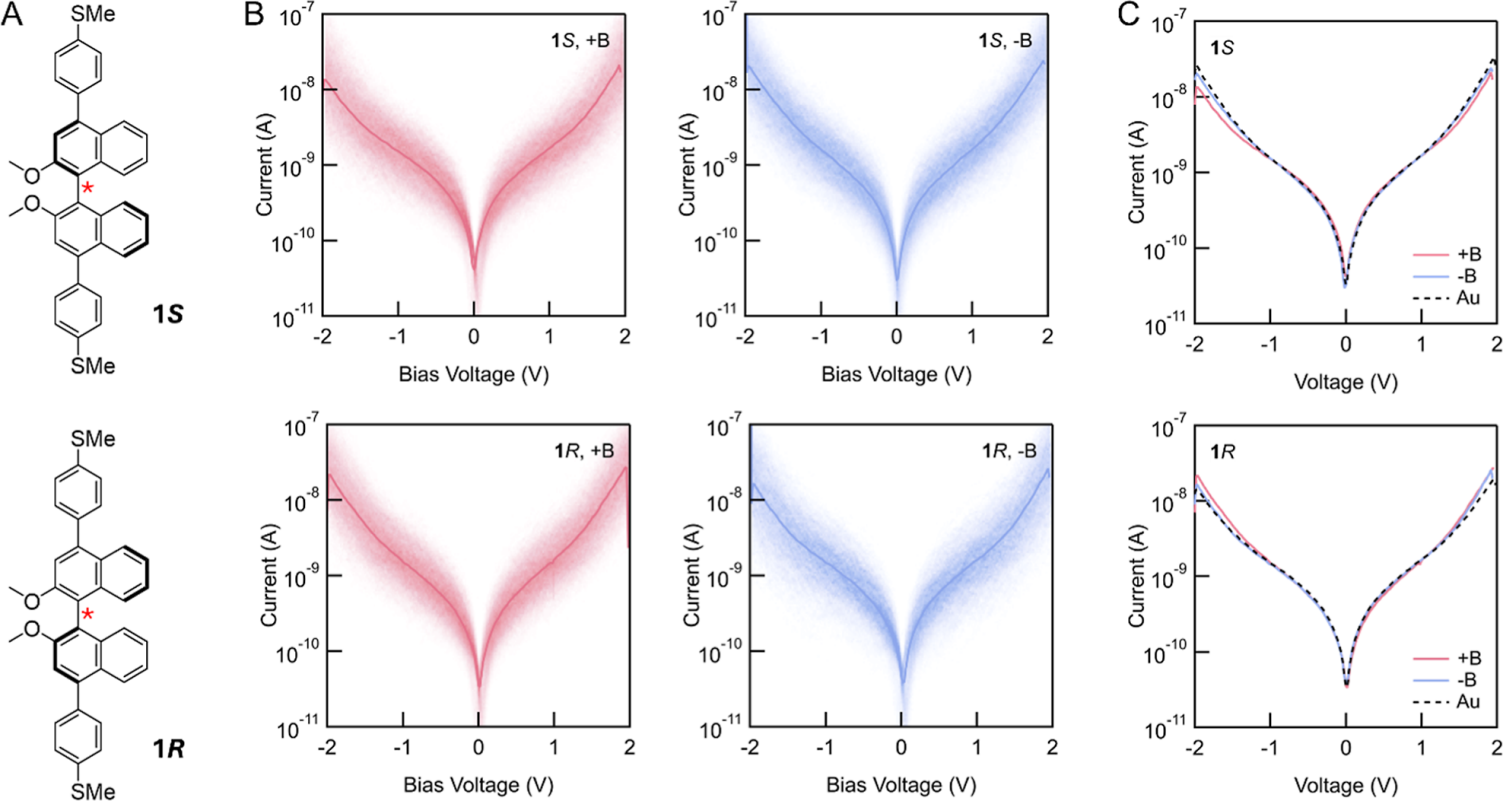
(A) Molecular structures of atropisomeric **1*S*** and **1*R***, with their stereogenic axes labeled by red stars. (B) 2D histogram of measured current–voltage traces of **1*S*** and **1*R*** junctions measured under external +B (red) and −B (blue) magnetic field; (**1*S***, +B) consists of 1256 traces selected from 22,000 measured traces; (**1*S***, −B) consists of 941 traces selected from 20,000 measured traces; (**1*R***, +B) consists of 1185 traces selected from 30,000 measured traces; and (**1*R***, −B) consists of 667 traces selected from 25,000 measured traces. (C) Average current–voltage curves of **1*S*** and **1*R*** under external +B (red) and −B (blue) magnetic field. The black dashed curves are analogous results obtained in the nonmagnetic STM-BJ setup using Au electrodes. The corresponding 2D histograms for the Au control measurements are shown in Supporting Information Figure S3.

We also measured the current–voltage characteristics of single-molecule junctions with **1** *S* and **1** *R* under external magnetic fields. In these experiments, we first pull the tip a fixed distance from the substrate, hold the distance fixed while the applied bias was swept between −2 and 2 V (see Supporting Information Section II for details) and then continue pulling the tip to break the junction. Current versus voltage data from traces that sustained a single molecule were selected from tens of thousands of measured traces for data analysis. These are presented as 2D current–voltage histograms in Figure 2B. The widths of the features in these 2D histograms indicates that the variation in measured current is approximately one-order of magnitude. We averaged each 2D histogram to obtain a single current–voltage trace for each measurement, and these traces are shown in Figure 2C for **1** ***S*** and **1** ***R***. The differences in the fitted current–voltage traces for **1** ***S*** and **1** ***R*** under opposite external magnetic fields are much smaller than the variation of current in individual measurements. In addition, we performed control current–voltage measurements for **1** ***S*** and **1** ***R*** using the nonmagnetic setup with a Au substrate. The current–voltage traces, shown as black dashed lines in Figure 2C (with the corresponding 2D histograms in Supporting Information Figure S4), align well with the traces from the magnetic measurements. We can conclude that there is no clear distinction between the behavior of ***1S*** and ***1R*** indicating that any spin filtering by these chiral molecules, if present, cannot be unambiguously determined from these results.

Next, we measured (2*S*,3*S*)- and (2*R*,3*R*)-bis­(diphenylphosphino)­butane molecules, labeled **2** ***S*** and **2** ***R*** in Figure 3A, obtained from Sigma-Aldrich. Measurements are made from TCB solution at 500 mV applied bias. The **2** ***S*** and **2** ***R*** molecules are different from **1** ***S*** and **1** ***R*** in two ways. First, **2** ***S*** and **2** ***R*** have aliphatic molecular backbones, thus electron transmission occurs through the σ-system instead of the π-system in **1** ***S*** and **1** ***R*** molecules. Second, **2** ***S*** and **2** ***R*** possess stereogenic centers, in contrast to the stereogenic axes in **1** ***S*** and **1** ***R***. This type of aliphatic chiral center has also been reported to show the CISS effect in both short nonhelical alanine-based molecules^59^ and long α-helix polyalanine wires.^23^ Similar to **1** ***S*** and **1** ***R***, **2** ***S*** and **2** ***R*** exhibit the same most probable conductance of 1.0 × 10^–2^ *G*_0_, as determined from the 1D conductance histograms (Figure 3B). Despite the small deviation of molecular conductance for **2** ***S*** and **2** ***R***, there is still no observable difference in their conductances under external magnetic fields of +B and −B. The resulting 2D histograms (Figure 3C) are almost identical across all measurements, showing flat molecular plateaus consistent with the electronic behaviors of molecular junctions formed by phosphino-linkers.^60^ The measurement results for **2** ***S*** and **2** ***R*** using the nonmagnetic setup with Au electrodes are shown in Supporting Information Figure S5, which shows the same most probable conductance as those obtained with the magnetic setup. Due to the short lengths of **2** ***S*** and **2** ***R***, we do not perform current–voltage measurement as it is challenging to hold the junction for the required time. However, the conductance measurement for numerous single-molecule junctions is sufficient to demonstrate that there is no observable spin-filtering effect in the coherent transport through **2** ***S*** and **2** ***R*** molecules.

**Figure 3:**
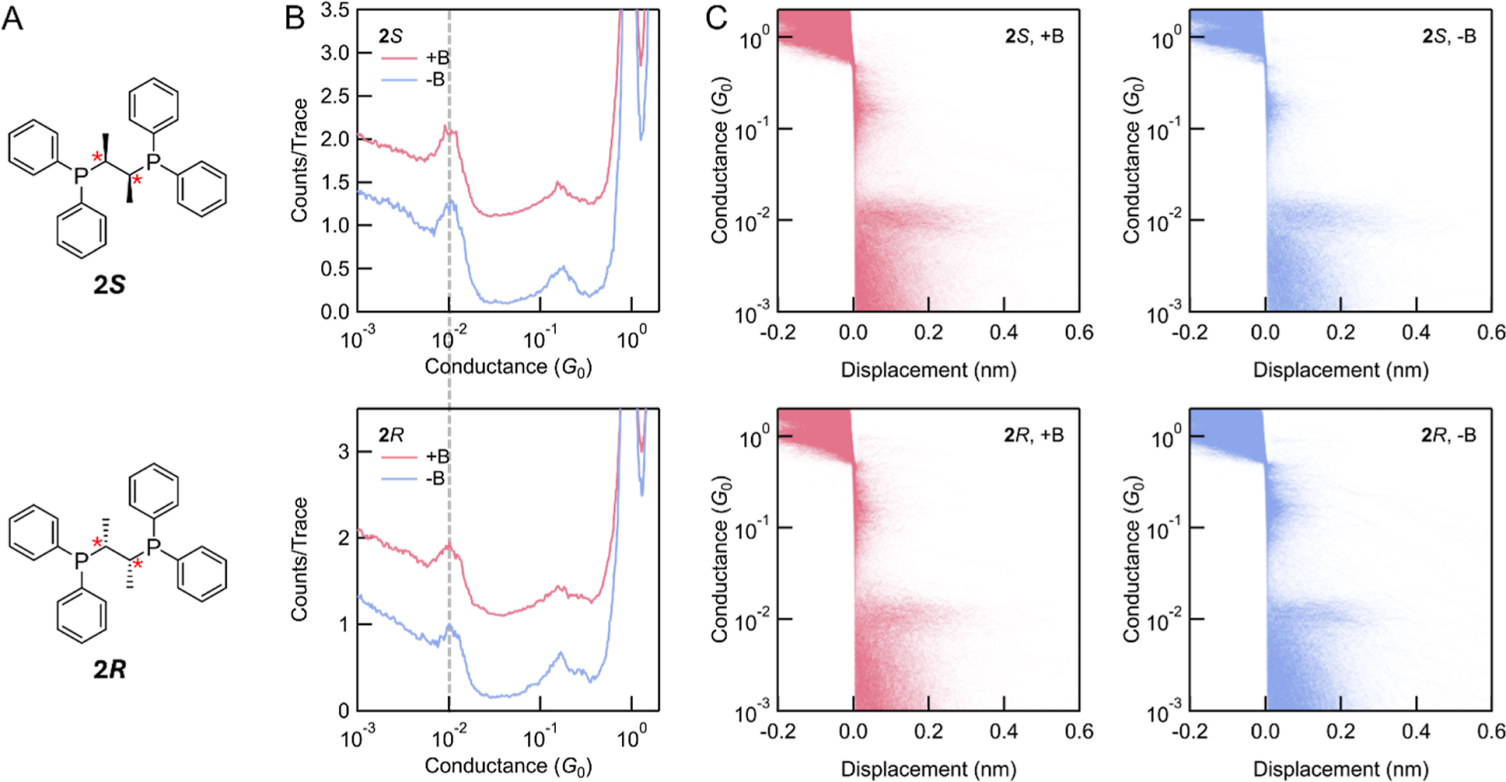
(A) Molecular structures of enantiomeric **2*S*** and **2*R***, with their stereogenic centers labeled with red stars. (B) 1D conductance histograms of **2*S*** and **2*R*** under +B and −B external magnetic fields; 8000 traces were measured for (**2*S***, +B); 4000 traces were measured for (**2*S***, -B); 5500 traces were measured for (**2*R***, +B); 6600 traces were measured for (**2*R***, −B). All the histograms are generated without data selection. The dashed line highlights the lack of shift in the conductance peak positions across the four measurements. (C) The corresponding 2D conductance-displacement histograms of **2*S*** and **2*R*** under +B and −B fields.

We next examined a subphthalocyanine molecule (**3**) illustrated in Figure 4A. Due to its contracted porphyrinic core coordinating a tetrahedral boron atom, this molecule exhibits a bowl-shaped geometry. With the three SEt substituents, this molecule poses chirality from its inherent geometric curvature, similar to helical molecules.^61^ Curvature has been shown to enhance effective SOC even in organic materials.^1,2,62^ This suggests that, this curved molecule could potentially exhibit a large spin polarization, as was seen in AFM-based measurements.^32^ We measured **3** in propylene carbonate (PC) solution at an applied bias of 100 mV. Molecule **3** has three identical aurophilic–SEt linkers, but we only observe a single conductance feature at 1.8 × 10^–4^ *G*_0_ in the 1D histogram (Figure 4B), which indicates that only two linkers participate in forming the single-molecule junction, each binding with one electrode. The 3-fold rotation symmetry of molecule **3** thus allows only one possible junction binding geometry. The 1D histograms show that molecular conductance is independent of the orientation of the magnetic field (+B and −B). The corresponding 2D conductance-displacement histograms (Figure 4C) are also identical under different fields and both show tilted molecular plateaus. A control measurement of **3** using the nonmagnetic setup (Supporting Information Figure S6) show the same conductance features. The measured conductances of **3** again do not provide any evidence of spin filtering in STM-BJ based transport measurements.

**Figure 4:**
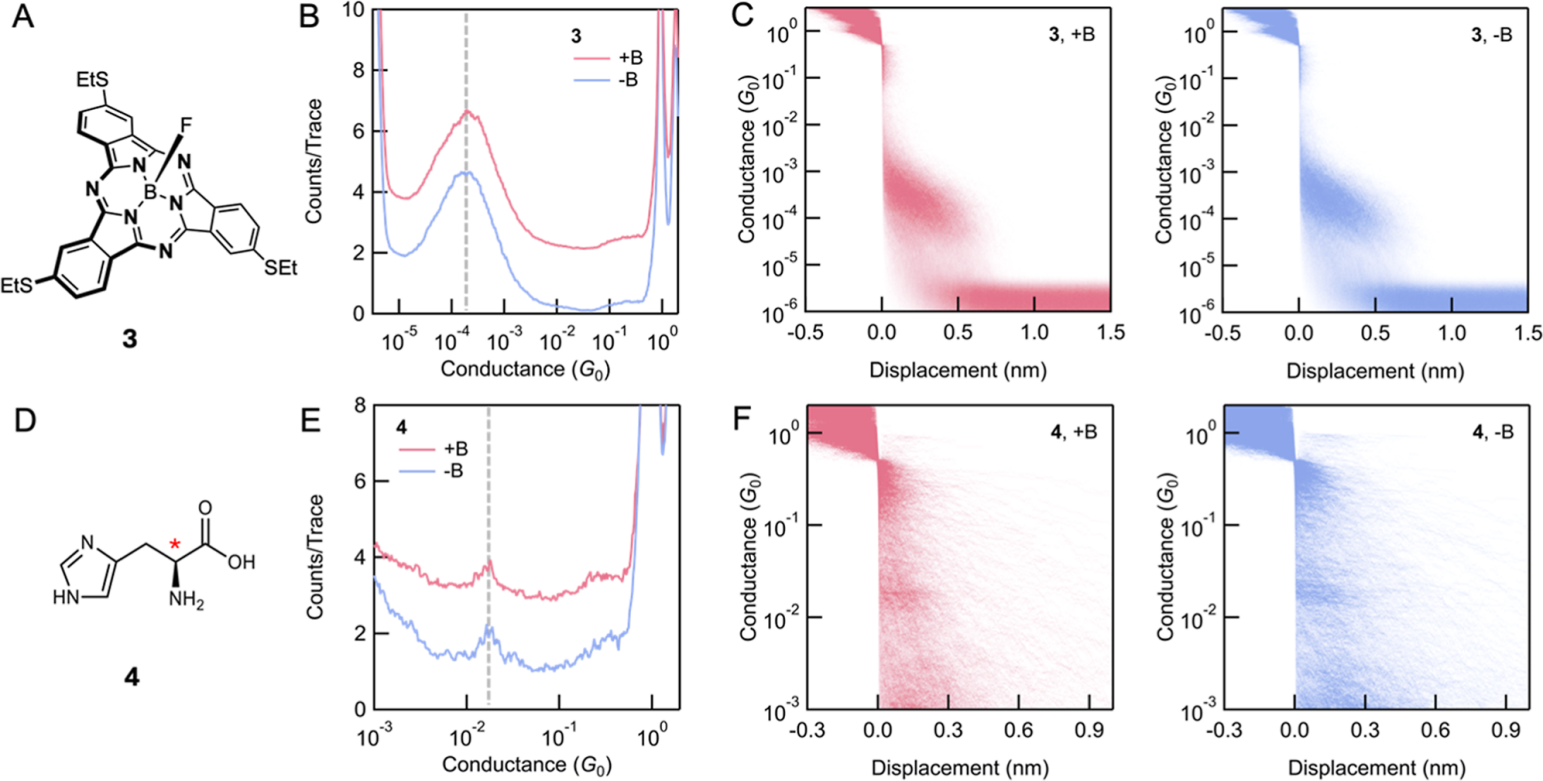
(A) Molecular structures of bowl-shaped **3**. (B) 1D conductance histograms of **3** under +B and −B external magnetic fields. 5000 traces were measured in both measurements and compiled into histograms without data selection. (C) The corresponding 2D conductance-displacement histograms of **3** under + B and −B fields. (D) Molecular structures of chiral biomolecule **4**. (E) 1D conductance histograms of **4** under +B and −B external magnetic fields. 3000 traces were measured in both measurements without data selection. (F) The corresponding 2D conductance-displacement histograms of **4** under +B and −B fields.

It is believed that molecular dipole moment in chiral molecules oriented along the electron-transport direction can enhance the chiral electrostatic potentials and chiral electric field **E**_chiral_, thereby generating a more significant CISS effect.^63,64^ To investigate this in single-molecule junctions, we measured an l-histidine amino acid, obtained from Sigma-Aldrich (**4**, Figure 4D), which has an intrinsic dipole moment of over 4 D, in water solutions under an applied bias of 500 mV using the magnetic setup. In the 1D conductance histograms (Figure 4E), we observed small but sharp molecular conductance peaks at 1.7 × 10^–2^ *G*_0_. The corresponding 2D conductance-displacement histograms (Figure 4F) showed short, flat conductance plateaus. The measured conductance value and the length of the molecular plateaus indicate that the molecular junctions are formed though the carboxylate and amino groups.^65^ If the orientation of the molecular dipole relative to the current direction were critical to observing the CISS effect, we would see a double peak in measurements of **4**. Furthermore, there is still no observed difference in molecular conductance under +B and −B fields, indicating again the absence of any significant spin filtering in coherent transport through molecular junctions. A control measurement of **4** using the nonmagnetic setup (Supporting Information Figure S7) show the same conductance features.

We now turn to theoretical calculations to shed light on the absence of CISS observed in our experiments. Various mechanisms have been proposed in the literature to explain the polarization reported across a wide range of systems, including spin–orbital angular momentum locking,^26,64,66,67^ electron correlation,^68^ and electron–phonon interactions.^27,28^ In our theoretical approach, we included a set of degrees of freedom and interactions we considered most relevant, with the goal of obtaining qualitative results. Specifically, we focused on modeling the SOC in the molecule, as this represents a critical component of many CISS mechanisms. We implemented a two-component approach within generalized Hartree–Fock theory to include SOC in our calculations using PySCF (see Supporting Information Section IV for details).^100^ We performed these calculations on an extended molecular system that included small gold clusters attached at both ends. Using the resulting effective one-electron Hamiltonian, projected onto the space of localized valence orbitals, we applied the Landauer approach to calculate transmission and spin polarization.

The calculated transmission and spin polarization for molecules **1** and **3** are shown in Figure 5. Our results indicate negligible spin polarization across a broad energy range near the Fermi energy consistent with the experimental results. We note that regardless of the placement of the position of the transport resonances relative to the Fermi level, the overall polarization remains extremely small even when compared with the scale of room-temperature fluctuations. This minimal polarization stems from the small ratio of on-site SOC to nearest-neighbor hopping elements in the Hamiltonian. For molecule **1**, analysis of the matrix elements between localized *p* carbon orbitals revealed that this ratio is of the order of 10^–3^. These calculations effectively rule out the mechanisms based on enhancement of SOC effects due to curved geometries. A calculation with a phenomenological treatment of weak electron–phonon coupling shows that it enhances the polarization by only up to a factor of 5 (see Supporting Information Section IV) and the absolute value remains insignificant. Although stronger electron–phonon coupling can lead to large spin polarization even with very small SOC, we do not expect polaronic effects to be relevant in the systems studied here. The presence of some gold atoms with strong atomic SOC also does not lead to substantial polarization in our calculations. Some theoretical models^14,69^ explain CISS by combining the substrate’s strong spin–orbit coupling with the orbital angular momentum locking found in chiral systems. Future studies will use first-principles calculations to more thoroughly investigate the feasibility of this mechanism. Overall, our calculations support the conclusion that in the coherent transmission regime of our experiments, the SOC in the organic system is insufficient to cause significant spin polarization.

**Figure 5:**
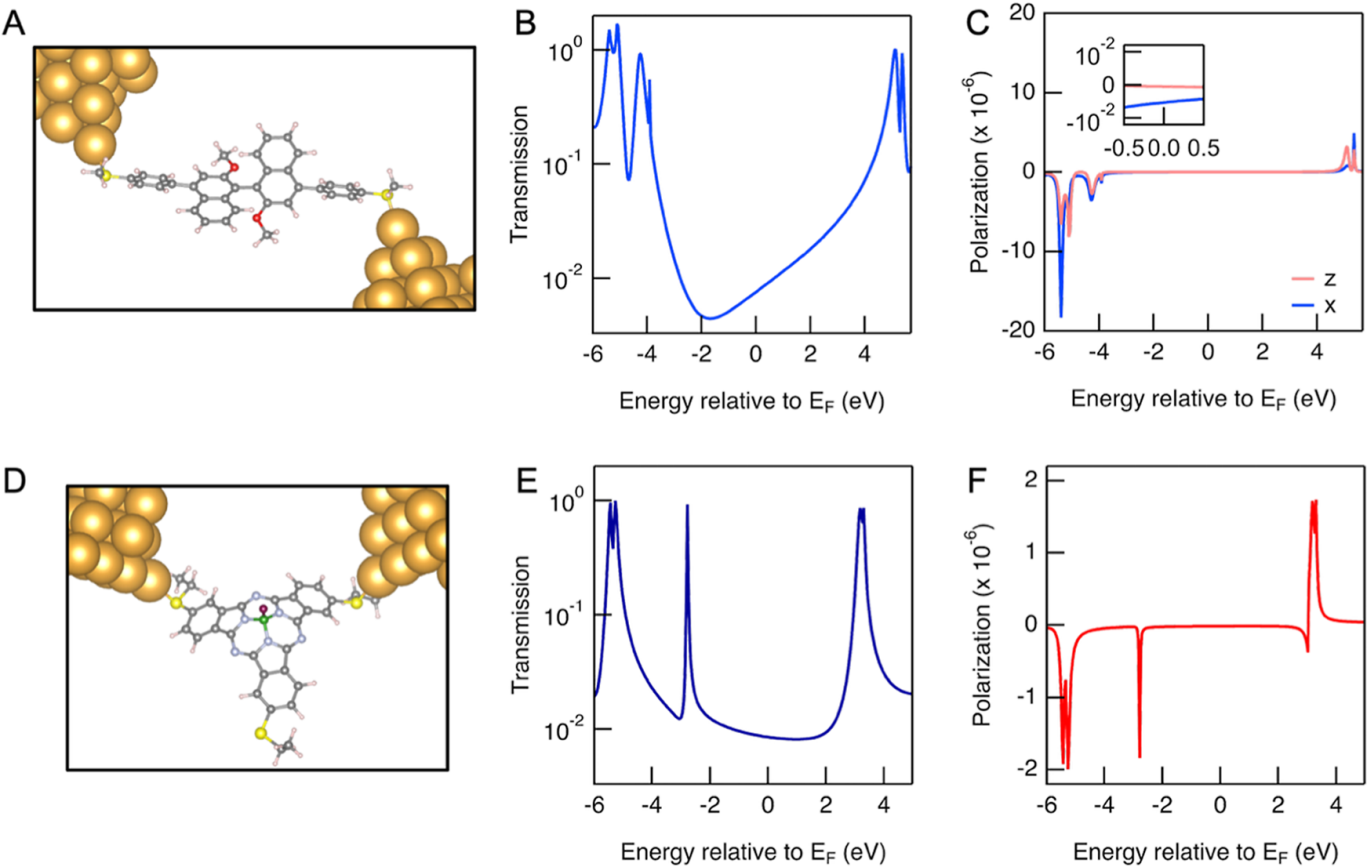
(A) Molecular junction structure for **1**. (B) Calculated transmission for junction shown in (A). (C) Calculated spin polarization as a function of energy for molecule **1** along the molecular axis (*z*) and transverse to the molecular axis (*x*). Inset shows the region around *E*_F_. (D) Molecular junction structure for **3**. (E) Calculated transmission for junction shown in D. (F) Calculated spin polarization as a function of energy for molecule **3**.

Based on both experimental and theoretical results, we conclude that there is no observable CISS effect that is clearly distinguishable in conductance and current versus voltage data in the coherent electron-transport regime in single-molecule measurements. We investigated chiral molecules with stereogenic axes (**1** ***S*** and **1** ***R***), stereogenic centers (**2** ***S*** and **2** ***R***), inherent bowl-shaped curvatures (**3**), and an electric dipole along electron-transport direction (**4**). The electronic properties of all the molecules are unaffected by the chirality of the molecules or by opposing external magnetic fields within the measurement distributions that we observe. Our results contradict conducting AFM based measurements that show large chirality-dependent currents even reaching 90% spin filtering.^22,30–33^ Although we do not have a clear explanation for this discrepancy, it could indicate that conducting AFM measurements are not in the coherent regime either due to the large number of molecules probed simultaneously or due to having multilayer molecular films. Our results do not contradict measurements at low temperatures under 2 T fields where a very small (<8%) effect at zero bias is observed.^70^ However, from an experimental perspective, to demonstrate conclusively that we see a CISS effect in the coherent transport regime would require changing the magnetic field while holding a molecule bound to two electrodes, and then repeating such a measurement thousands of times to obtain statistically relevant data. Such an experiment would have complications relating to thermal currents induced by changing magnetic fields and would also not achievable at room temperature.

Our experimental results supported by our theoretical findings highlight the fact that within a coherent transport regime, the probability of a spin-flipping transition is below 10^–6^, making the effect too small to observe. Our findings indicate that the manifestation of the CISS effect is highly dependent on specific systems and conditions. Within the experimental and theoretical frameworks of this study, clear evidence for CISS remains elusive.

## Supplementary Material



## Notes

Synthetic, measurement and theoretical procedures and additional data (PDF)

## ○

L. L. Department of Chemistry, Massachusetts Institute of Technology, Cambridge, MA 02139

## ⧫

J. L. Department of Molecular Engineering, Kyoto University Katsura, Nishikyo-ku, Kyoto 615–8510, Japan.
