# Supplementary material for: Too Fast for Spin Flipping: Absence of Chirality-Induced Spin Selectivity in Coherent Electron Transport through Single-Molecule Junctions
Source: J Am Chem Soc. 2025 Jul 2;147(28):25043–51. doi: 10.1021/jacs.5c08517 (PMC12272679; doi:10.1021/jacs.5c08517)
Supplement: Supplementary file 1 [file ja5c08517_si_001.pdf]

Supporting Information for

Too Fast for Spin Flipping: Absence of Chirality-Induced Spin Selectivity in Coherent Electron Transport through Single-Molecule Junctions

Liang Li[1], Wanzhuo Shi[1], Ankit Mahajan[1], Junxiang Zhang[2], Marta Gómez-Gómez[3], Jorge Labella[3], Shayan Louie[1], Tomás Torres[3,4,5*], Stephen Barlow,[6] Seth R. Marder[2,6*], David R. Reichman[1*], Latha Venkataraman[1,7,8*]

[1]Department of Chemistry, Columbia University, New York, NY 10027, United States

[2]Renewable and Sustainable Energy Institute (RASEI), University of Colorado Boulder, Boulder, CO 80309, USA.

[3] Department of Organic Chemistry. Universidad Autónoma de Madrid, Campus de Cantoblanco, C/Francisco Tomás y Valiente 7, 28049 Madrid, Spain

[4] Institute for Advanced Research in Chemical Sciences (IAdChem), Universidad Autónoma de Madrid, 28049 Madrid, Spain

[5] IMDEA-Nanociencia, Campus de Cantoblanco, 28049 Madrid, Spain

[6]Departments of Chemical and Biological Chemistry and of Chemistry, University of Colorado Boulder, Boulder, CO 80309, USA.

[7]Department of Applied Physics and Applied Mathematics, Columbia University, New York, NY 10027, United States

[8]Institute of Science and Technology Austria, 3400 Klosterneuburg, Austria

Table of Contents:

I. **Syntheses**

II. **STM-BJ Measurements**

III. **DFT Calculations**

IV. **Ab Initio Calculations**

V. **References**

I. Syntheses

Syntheses of **1*S*** and **1*R***.

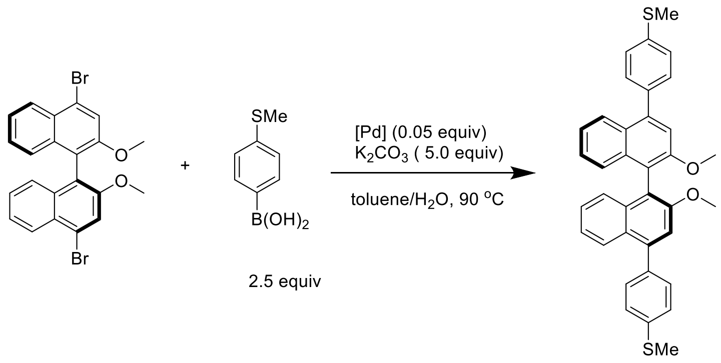

The synthesis of **1*S*** was achieved with a modified reported procedure.[1] Under the argon atmosphere, a 3-neck reaction flask was charged with 4- (methylthio)phenylboronic acid (538 mg, 3.2 mmol, 2.5 eq), (1*S*)-4,4′-dibromo-2,2′-dimethoxy-1,1′-binaphthalene (610 mg, 1.3 mmol, 1.0 eq), potassium carbonate (883 mg, 6.4 mmol, 5 eq), and [1,1′-bis(diphenylphosphino)ferrocene]dichloropalladium dichloromethane (53 mg, 0.07 mmol, 5 mol%). The solids were degassed under vacuum and the flask was refilled with Ar. In a separate flask was added toluene (12 mL) and DI water (2 mL), and these were sparged with Ar for 30 min. The solvents were transferred to the 3-neck reaction flask via cannula transfer and the resulting mixture was stirred at 90 °C for 20 hrs. After cooling to room temperature, the reaction mixture was extracted with toluene and washed with aqueous NH_4Cl solution. The organic layer was concentrated and further purified by silica gel column chromatography using hexanes:DCM (1:1) as the eluent to yield **1*S*** as a highly blue-fluorescent white solid (660 mg, 91%). **1*S*** dissolves well in $CHCl_3$, DCM, toluene, etc. ^1H NMR (400 MHz, $CDCl_3$) δ 8.07 – 7.85 (m, 2H), 7.71 – 7.56 (m, 4H), 7.51 – 7.45 (m, 4H), 7.42 (s, 2H), 7.35 – 7.26 (m, 6H), 3.84 (s, 6H), 2.63 (s, 6H). $^{13}C\{^1H\}$ NMR (101 MHz, $CDCl_3$) δ 154.41, 141.18, 137.79, 137.72, 134.52, 130.64, 127.55, 126.41, 126.31, 126.13, 125.75, 123.67, 119.08, 115.34, 56.96, 15.89. HRMS (ESI) calcd for $C_{36}H_{31}O_2S_2$ ($(M+H)^+$), 559.1760; found, 559.1773. Anal. Calcd. for $C_{36}H_{30}O_2S_2$: C, 77.39; H, 5.41. Found: C, 77.31; H, 5.25.

For **1*R***, the same precedure was used except the starting material, (1*R*)-4,4′-dibromo-2,2′-dimethoxy-1,1′-binaphthalene. The reaction yield is 93%. ^1H NMR (400 MHz, $CDCl_3$) δ 8.06 –

7.88 (m, 2H), 7.72 – 7.58 (m, 4H), 7.52 – 7.45 (m, 4H), 7.42 (s, 2H), 7.35 – 7.26 (m, 6H), 3.84 (s, 6H), 2.63 (s, 6H). $^{13}C\{^1H\}$ NMR (101 MHz, CDCl3) δ 154.41, 141.18, 137.80, 137.72, 134.52, 130.65, 127.55, 126.41, 126.31, 126.13, 125.75, 123.67, 119.07, 115.33, 56.96, 15.89. HRMS (ESI) calcd for $C_{36}H_{31}O_2S_2$ ($(M+H)^+$), 559.1760; found, 559.1767. Anal. Calcd. for $C_{36}H_{30}O_2S_2$: C, 77.39; H, 5.41. Found: C, 77.00; H, 5.43.

Photophysical properties of **1*S*** and **1*R*** are shown in Figure S1.

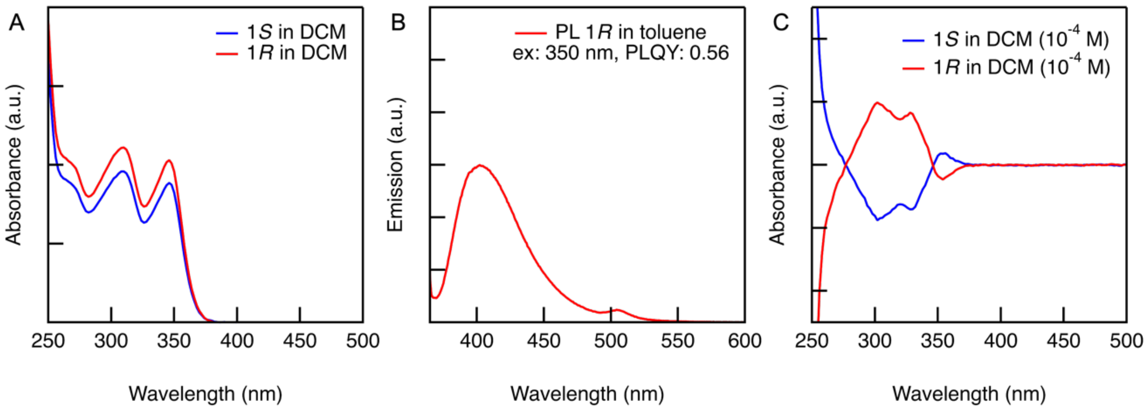

*Figure S1. A. Absorption spectra of **1S** and **1R** in the diluted DCM solution. B. Emission spectrum of **1R** in the diluted toluene solution (PLQY: ~0.56). C. Circular dichroism (CD) spectra of **1S** and **1R** in DCM solution (~10^{-4} M).*

NMR spectra

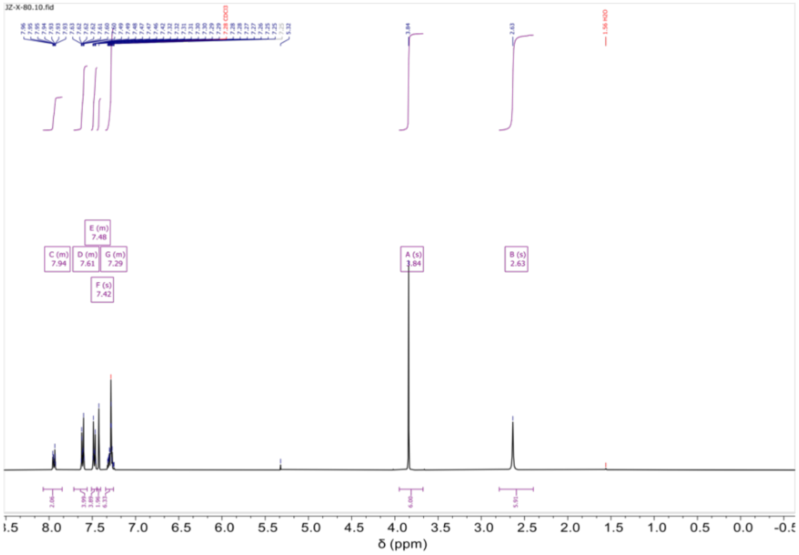

*^1H NMR spectrum of **1S** in $CDCl_3$.*

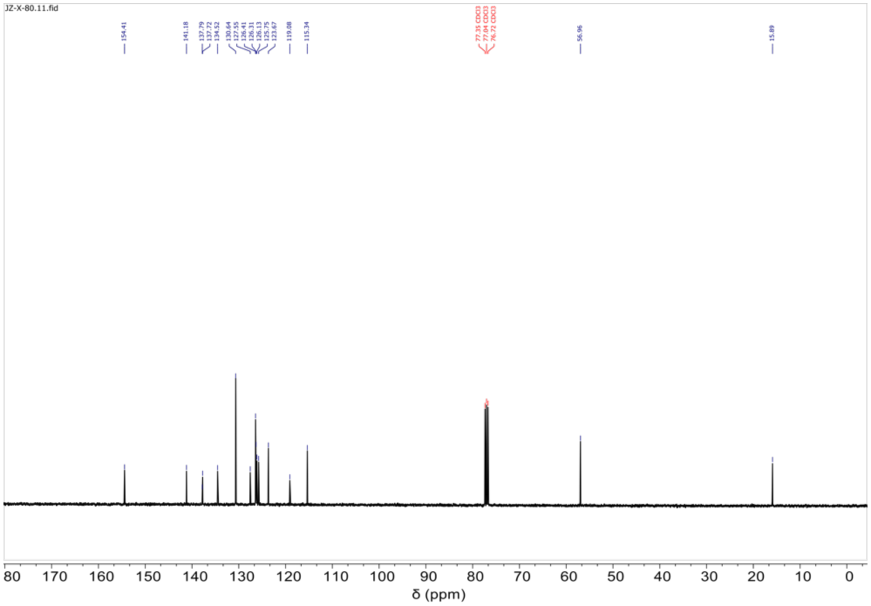

$^{13}C\{^{1}H\}$ *NMR spectrum of* ***1S*** *in* $CDCl_3$.

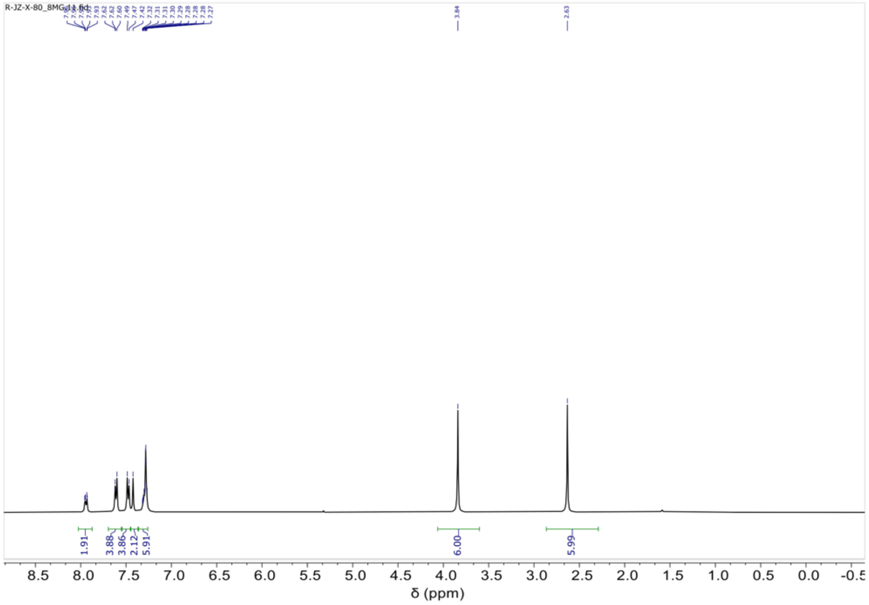

^{1}H *NMR spectrum of* ***1R*** *in* $CDCl_3$.

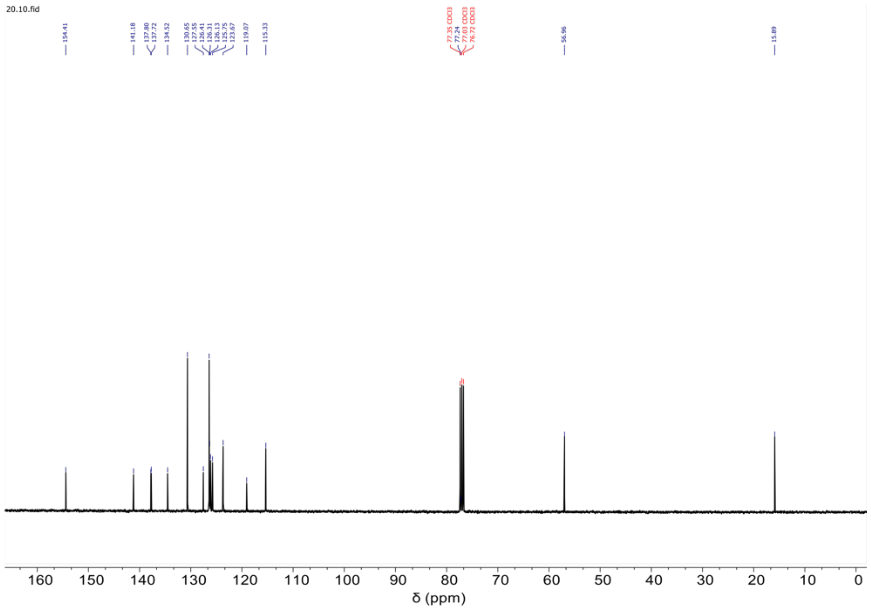

*$^{13}C\{^1H\}$ NMR spectrum of **1R** in $CDCl_3$.*

Synthesis of 3

The monitoring of the reactions has been carried out by thin layer chromatography (TLC), employing aluminum sheets coated with silica gel type 60 F254 (0.2 mm thick, Merck). The analysis of the TLCs was carried out with an UV lamp of 254 and 365 nm. Purification and separation of the synthesized products were performed by normal-phase column chromatography, using silica-gel 60 (230–400 mesh, 0.040–0.063 mm, Merck) as the stationary phase. Eluents along with the relative ratio in the case of solvent mixtures are indicated for each particular case. Nuclear magnetic resonance spectra (^{1}H-, ^{13}C-,^{11}B-, ^{19}F-NMR) were recorded on a Bruker AV-300 or Bruker DRX-500 spectrometers. Deuterated solvent employed in each case is indicated in brackets, and its residual peak was used to calibrate the spectra using literature reference δ ppm values.[2] All the experiments were recorded at room temperature. Multiplicity was indicated using the following abbreviations: dd (doublet of doublets), t (triplet), m (multiplet). High-resolution mass spectra (HR-MS) were recorded in the Interdepartmental Investigation Service of UAM, employing matrix-assisted laser desorption/ionization time-of-flight (MALDI-TOF) using a Bruker-Ultraflex-III spectrometer with a Nd:YAG laser operating at 355 nm or ultrafleXtreme spectrometer. The matrixes and internal references employed are indicated for each spectrum. Mass spectrometry data are expressed in m/z units. Ultraviolet and visible (UV-vis) spectra were recorded using solvents in the spectroscopic grade in the Organic Chemistry Department of UAM employing a

JASCO-V660 spectrophotometer. All reactions dealing with air or moisture sensitive compounds were carried out by standard Schlenk techniques in a dry reaction vessel under argon. Chemicals were purchased from commercial suppliers and used without further purification. Dry solvents were purchased from commercial suppliers in anhydrous grade or thoroughly dried before use, employing standard methods. Solid, hygroscopic reagents were dried in a vacuum oven before use.

The resolution of **3** was carried out by High Performance Liquid Chromatography (HPLC) using a Shimadzu equipment with a preparative Daicel Chiralpak IC column (3 cm ø x 25 cm 5 µm). The enantiopurity was then checked on an Agilent 1200 equipment with a semi-preparative Daicel Chiralpak IC column (10 mm ø x 20 mm). The separation conditions are indicated in each case.

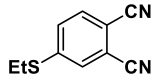

4-(ethylthio)phthalonitrile

The synthesis and characterization of **4-(ethylthio)phthalonitrile** have been previously reported.[3]

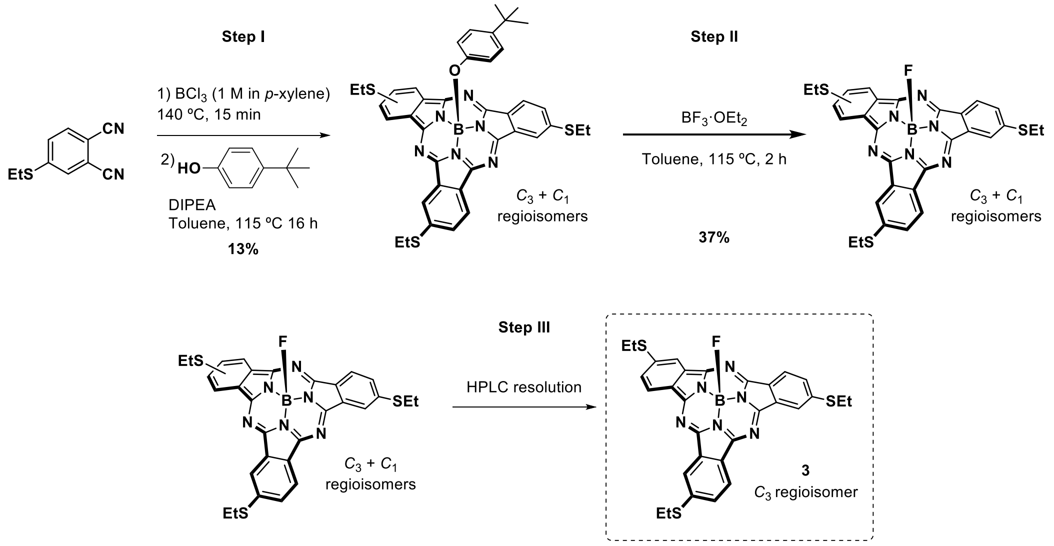

Step I: In a 50 mL two-necked round-bottomed flask, equipped with a condenser, magnetic stirrer and rubber seal, a 1.0 M solution of BCl_3 in *p*-xylene (5.3 mL) was added over 4-(ethylthio)phthalonitrile (1 g, 5.3 mmol) under argon atmosphere. The reaction mixture was stirred at 140 ºC for 15 min. The purple solution was allowed to cool to room temperature. Then, it was flushed with argon to remove the excess of BCl_3. After removal of the solvent, 4-*tert*-butylphenol (1.9 g, 12.6 mmol), DIPEA (*N*,*N*-diisopropylethylamine; 0.23 mL, 1.8 mmol) and dry toluene (5.3

mL) were added over the crude under argon atmosphere. The new reaction mixture was stirred at 115 °C for 16 h. The dark purple reaction slurry was dissolved in toluene/AcOEt (10:1) and passed through a short silica plug. The solvent was removed by vacuum distillation and the resulting dark solid was purified by column chromatography on silica gel using AcOEt/heptane 1:2 as eluent. Since C_{2v}-symmetric phthalonitriles lead to the formation of a mixture of C_1-symmetric and C_3-symmetric SubPc in a statistical 1:3 C_3:C_1 ratio,[4] the resulting solid is composed of both reigioisomers of the SubPc bearing axial 4-*tert*-butylphenoxy, which was precipitated in a DCM/MeOH mixture.

Step II: In a 25 mL Schlenk tube, the resulting purple powder (163.6 mg, 0.23 mmol), $BF_3{\cdot}OEt_2$ (0.14 mL, 1.15 mmol) and dry toluene (1.6 mL) were added under argon atmosphere. The reaction mixture was stirred at 115 ºC for 2 h. The purple-blue solution was allowed to cool to room temperature and diluted with toluene (4 mL). Then, pyridine was added dropwise until the color of the reaction returned to a characteristic SubPc purple color. The purple solution was passed through a short silica plug (eluent: toluene/AcOEt 3:1). The solvent was removed by vacuum distillation. The product was precipitated in a DCM/MeOH mixture, obtaining a purple powder composed of a C_3+C_1 regioisomers mixture of the SubPc **3**.

Step III: Compound **3** (C_3 regioisomer) was isolated from C_1 regioisomer and obtained directly as an enantiopure compound via HPLC resolution. The product was precipitated in a DCM/MeOH mixture to give **3** as a dark purple solid.

^{1}H-NMR (300 MHz, $CDCl_3$): δ (ppm) = 8.71-8.69 (m, 6H), 7.79 (dd, $^3J_{H\text{-}H}$ = 8.5 Hz, $^4J_{H\text{-}H}$ = 1.6 Hz, 3H), 3.31-3.19 (m, 6H), 1.47 (t, $^3J_{H\text{-}H}$ = 7.4 Hz, 9H); **^{13}C-NMR** (75 MHz, $CDCl_3$) δ (ppm) = 151.2, 150.7, 141.3, 132.0, 129.70, 128.3, 122.4, 120.1, 27.4, 14.2; **^{11}B-NMR** (96 MHz, $CDCl_3$) δ (ppm) = -14.0 (d, J = 31 Hz, 1B; B-F); **^{19}F-NMR** (282 MHz, $CDCl_3$) δ (ppm) = -156.7 (q, J = 20.0 Hz, 1F; B-F); **UV-vis** (THF): λ (nm) (log ε/dm^3 mol^{-1} cm^{-1}) = 582 (4.9), 538 (sh), 363 (4.4); **HR-MS** (MALDI-TOF): Calculated for $C_{30}H_{24}BFN_6S_3$: 594.1302, found: 594.1309; **Mp** > 250 ºC.

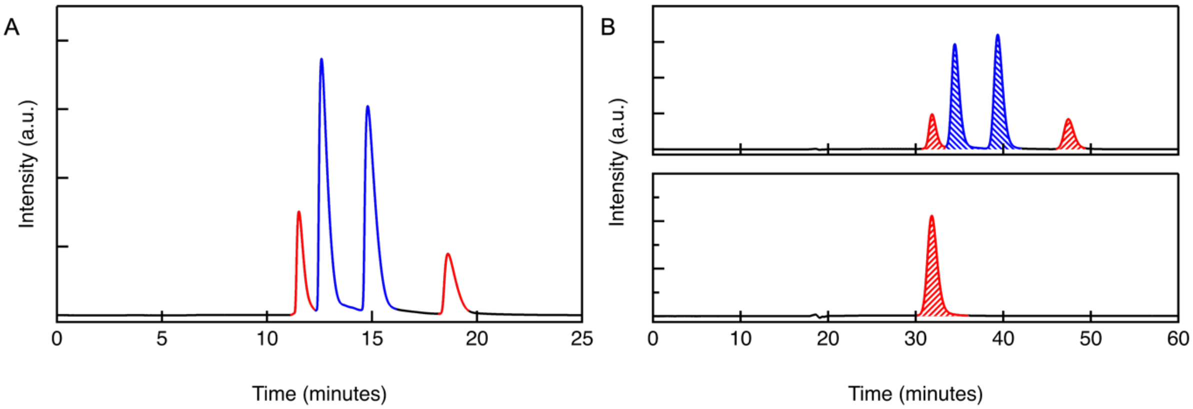

*Figure S2. HPLC chromatogram of C_3+C_1 regioisomers resolved in Step III (A, preparative isolation) and HPLC chromatogram to check the purity of **3** (B, semi-preparative isolation), with peaks corresponding to both enantiomers of C_3 (red traces) and C_1 reigioisomers (blue traces). The percentage area underneath the peaks is 12.5%, 37.5%, 37.5% and 12.5%, respectively. A) Eluting solvents is a mixture of dichoromethane and n-hexane in 80:20 ratio. Flow rate is 20.0 mL min^{-1} in A and 1.0 mL min^{-1} in B. Detection wavelength is 570 nm.*

NMR spectra

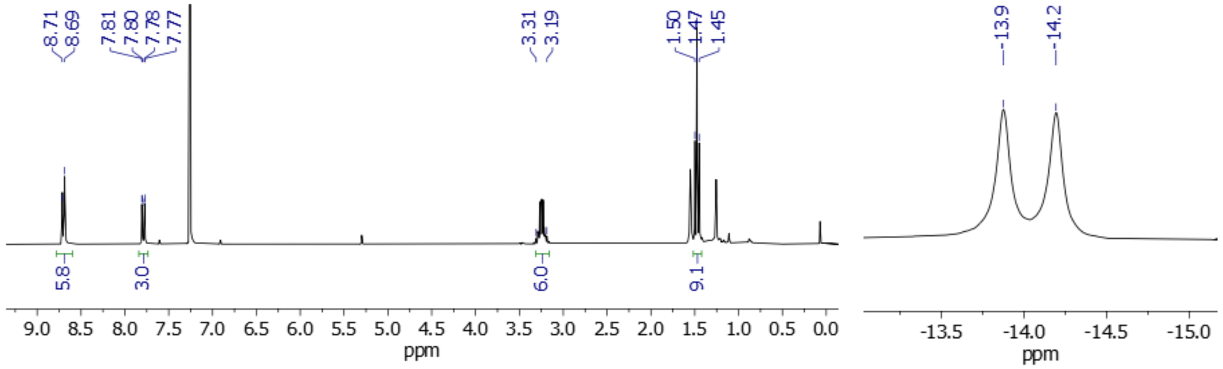

*^{1}H-NMR (left) and ^{11}B-NMR (right) spectrum ($CDCl_3$) of **3**.*

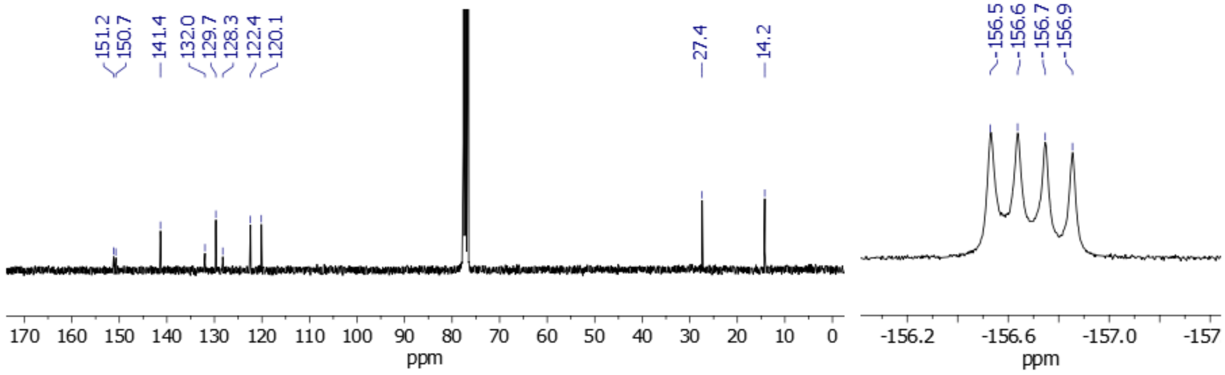

*^{13}C-NMR (left) and ^{19}F-NMR (right) spectrum ($CDCl_3$) of **3**.*

MALDI-TOF spectra

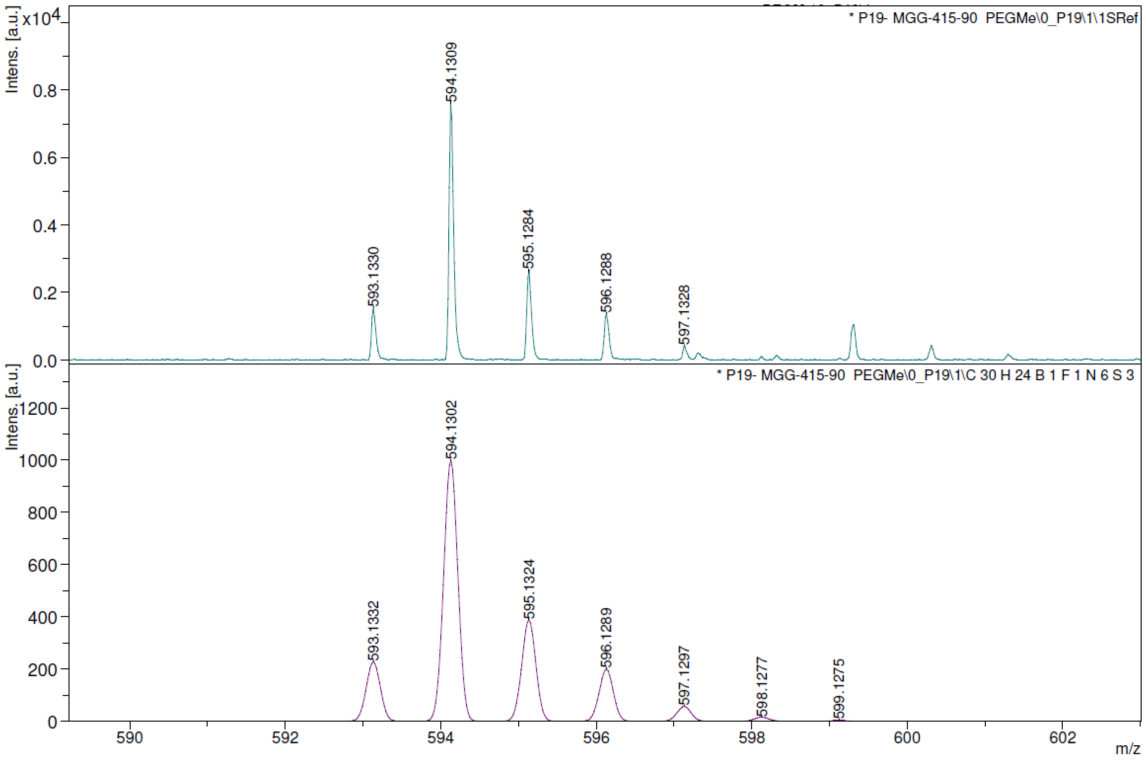

MALDI-TOF mass spectrum of ***3*.**

II. STM-BJ Measurements

Conductance Measurements:

STM-BJ conductance measurements were carried out using a custom-built Scanning Tunneling Microscope (STM)[5]. We used 0.25 mm diameter cut gold wire (99.95%, Alfa Aesar) as the STM tip. For the non-magnetic setup, we used ~100 nm thick gold-coated (99.999%, Alfa Aesar) steel pucks as the substrates, and the gold substrates were UV/ozone cleaned for 20 minutes before use. For the magnetic setup, we used a hetero-structured Si/Ti(10 nm)/Ni(100 nm)/Au(8 nm) as the substrates with a NdFeB permanent magnet at the bottom to magnetize the Ni layer. The substrates were change to new ones when flipping the external magnetic field to reset the measuring conditions. A commercially available single-axis piezoelectric positioner (P-840.10, PI) was used to control the tip-substrate distance at sub-angstrom level. The STM setup was controlled using custom software written in IgorPro (Wavemetrics, Inc.) and operated under ambient conditions at room temperature. For measurements in 1,2,4-trichlorobenzene (TCB) solutions (molecule **1*S***, **1*R***, **2*S***, and **2*R***), we used plain cut gold wire as the gold tip. For measurements in propylene carbonate (PC) and water solutions (molecule **3** and **4**), the gold tip is coated by Apiezon wax to minimize background current in these polar solutions. The concentration of the molecular solutions is 0.1 mM. After the formation of each gold-gold contact junction with a conductance greater than $5G_0$, the piezoelectric positioner moved the tip at a speed of 20 nm/s to break the junction. The current and voltage across the junction were measured at 40 kHz with a voltage applied across the junction in series with a 100 kΩ resistor to avoid saturating the current amplifier. During the retraction of the STM tip, a gold point contact is formed with a conductance close to $1G_0$. When the gold contact is broken with the presence of solutions of molecules with gold-binding groups, molecular conductance plateaus are observed below $1G_0$. The measured conductance (current/voltage) traces were then collected and compiled into logarithmically binned 1D conductance histograms, while 2D conductance-displacement histograms were obtained by overlaying all the measured traces after aligning them at a conductance of $0.5G_0$.

Current-Voltage Measurements

Current-voltage measurements were performed by firstly withdrawing the gold tip at 20 nm/s to form a gap between the tip and the substrate at an applied bias. Next, the tip is held at a fixed position when the external bias is ramped continuously between -2 V and +2 V for one cycle.

Then, the tip was further withdrawn at 20 nm/s to break the junction. Traces were selected where the conductance at the beginning and at the end of the holding section is between $10^{-4}G_0$ to $10^{-6}G_0$ for **1*S*** and **1*R*** and were also selected for traces without capacitive current at zero bias. The 2D current-voltage histograms were constructed from the hold section of the selected traces using logarithmically binned current and linearly binned voltage.

Supplementary Figures for STM-BJ Measurements

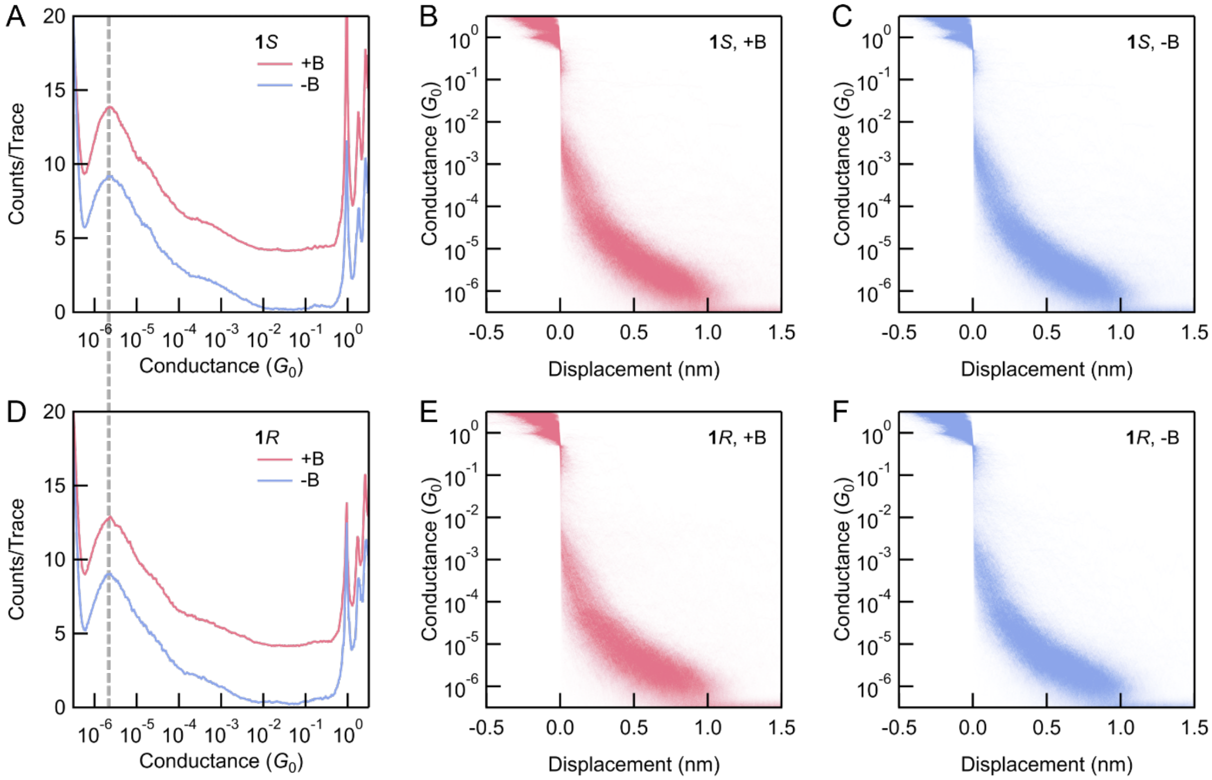

*Figure S3. (A) 1D conductance histograms of **1S** under +B and -B external magnetic fields. 3000 traces were measured for both measurements without data selection. (B-C) The corresponding 2D conductance-displacement histograms of **1S** under +B and -B fields, respectively. (D) 1D conductance histograms of **1R** under +B and -B external magnetic fields. 3000 traces were measured for both measurements without data selection. (E-F) The corresponding 2D conductance-displacement histograms of **1R** under +B and -B fields, respectively.*

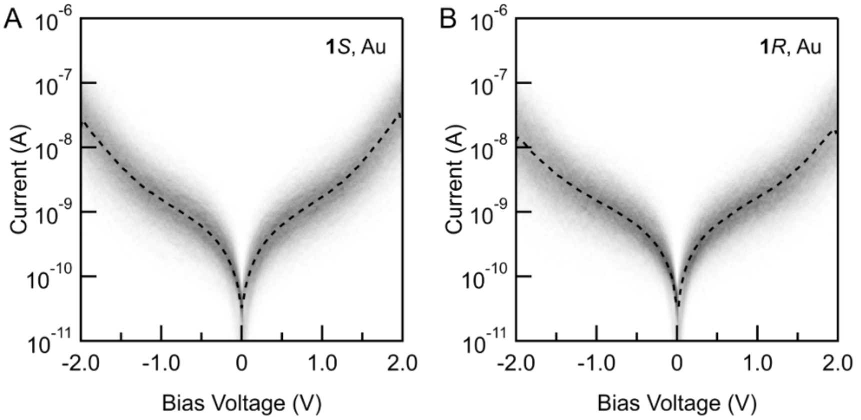

Figure S4. Current-Voltage measurement of (A) ***1S*** *and (B)* ***1R*** *using the non-magnetic setup with gold electrodes. (A) consists of 1791 traces selected from 30000 measured traces. (B) consists of 1679 traces selected from 29000 measured traces.*

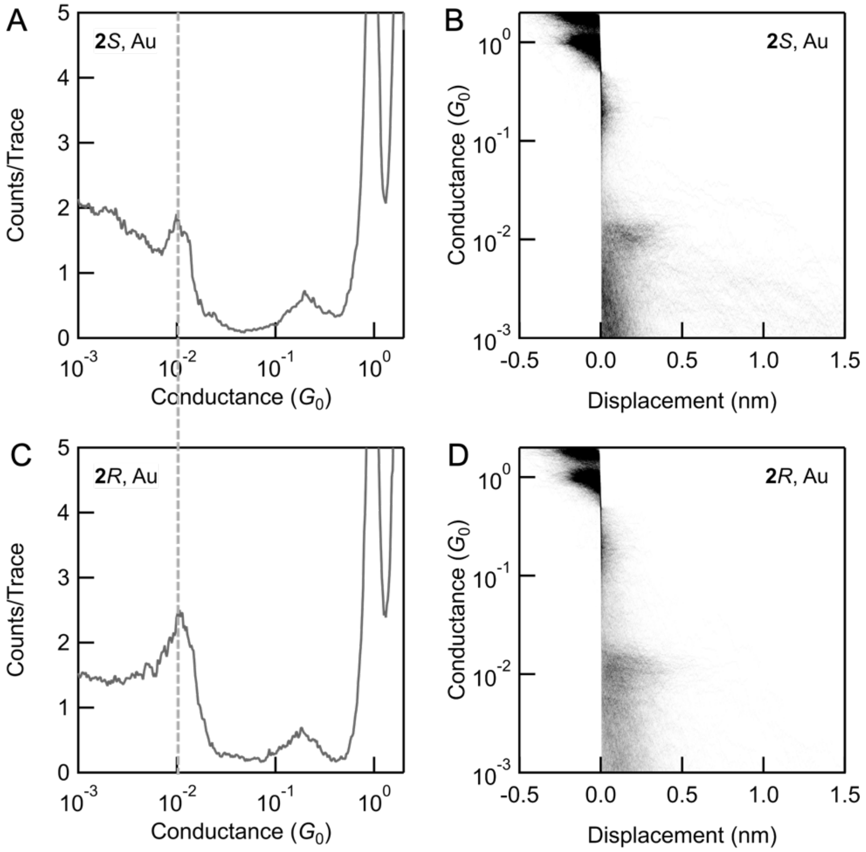

Figure S5. Au control measurements of ***2S*** *and* ***2R*** *using the non-magnetic setup. (A) 1D histogram and (B) 2D histogram of* ***2S****. (C) 1D histogram and (D) 2D histogram of* ***2R****. 4000 traces were collected for both measurements without data selection.*

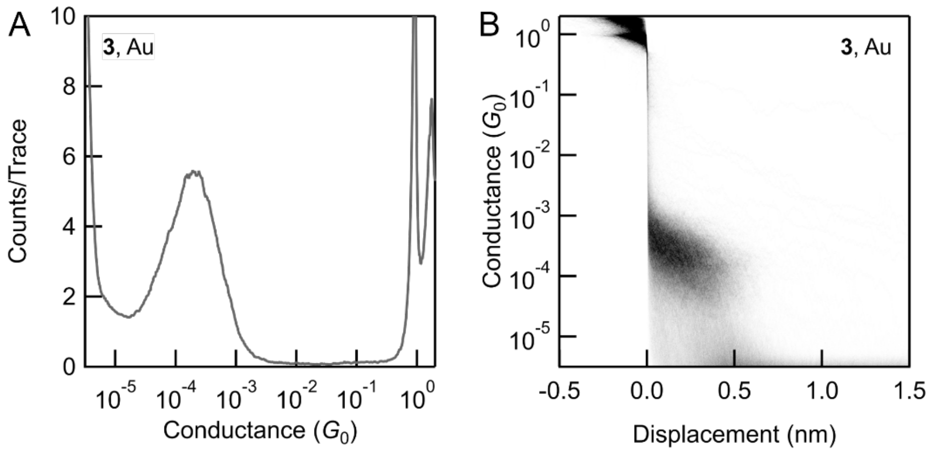

*Figure S6. Au control measurements of **3** using the non-magnetic setup. (A) 1D histogram and (B) 2D histogram of **3**. 5000 traces were collected without data selection.*

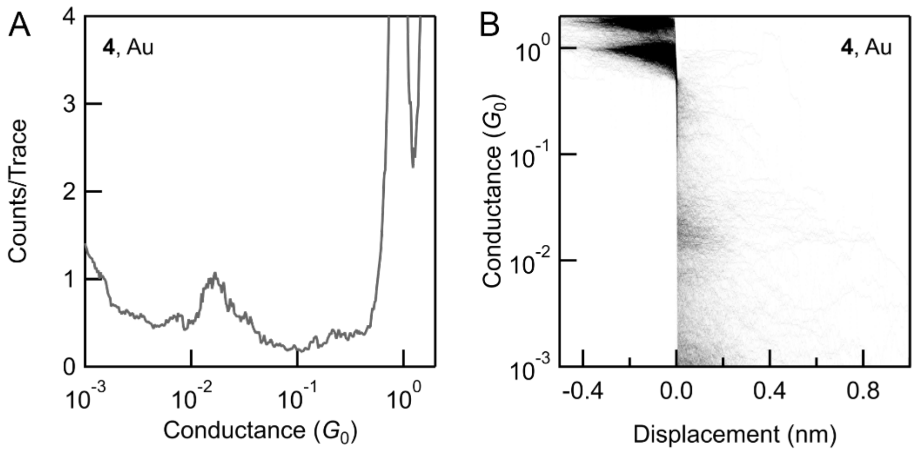

*Figure S7. Au control measurements of **4** using the non-magnetic setup. (A) 1D histogram and (B) 2D histogram of **4**. 3000 traces were collected without data selection.*

III. DFT Calculations

Kohn-Sham Densities of States

The Kohn-Sham Densities of States (KS DOS) were calculated by FHI-aims software[6] using a non-empirical generalized gradient-corrected approximation (Perdew-Burke-Ernzerhof, PBE) for the exchange-correlation functional[7]. We incorporated the scalar relativistic corrections to the kinetic energy into the first-principles calculations at the atomic zeroth-order regular approximation (ZORA) level[8]. The Kohn-Sham states were represented in an optimized all-electron numeric atom-centered basis set with "light" computational settings. The DOS was calculated from an integration of δ-functions within the Brillouin zone in k-space,

$$g(\varepsilon) = \sum_n \int d^3k\, \delta\big(\varepsilon - \varepsilon_n(\mathbf{k})\big), \tag{1}$$

where n is the band index and $\mathbf{k}$ is the crystal momentum. In our calculation, we replace the δ-functions with Gaussian functions of width 0.05 eV to avoid singularities. We calculated the KS DOS for both face-centered cubic Ni (magnetized and demagnetized, Figure 1C in the main article) and face-centered cubic Au (Figure S6) crystals. The calculation results were obtained using the convergence criteria in the self-consistent field cycle for the difference in the particle density (10^{-5} electrons/Å^3), total energy (10^{-5} eV), sum for of the Kohn-Sham eigenvalues (10^{-5} eV) and forces (10^{-4} eV/Å).

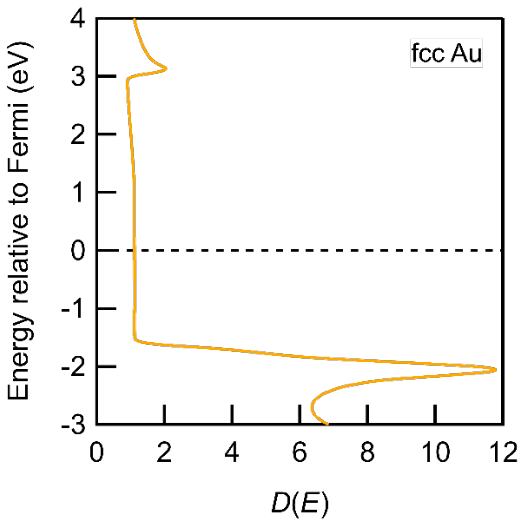

Figure S8. Calculated KS DOS for face-centered cubic Au.

IV. Ab Initio Calculations

1) Ab Initio Calculation of Spin-Orbit Coupling

We use the exact two component (X2C) method[9] to calculate the spin-orbit coupling (SOC) matrix elements. The X2C Hamiltonian is obtained by a unitary transformation of the Dirac Hamiltonian which decouples the four-component Dirac bispinor into small and large spinors. The resulting Hamiltonian for the relevant large component includes both spin-free and spin-dependent relativistic terms. The one-electron terms are given by

$$H^{1}_{\mathrm{X2C}} = h_{ij} a_i^\dagger a_j \tag{3}$$

where $a^\dagger$ and a are creation and annihilation operators, i and j are spin orbitals, and h_{ij} are complex valued matrix elements. We use the generalized Hartree Fock (GHF) method to obtain the one-electron spectra. We obtain effective Hamiltonians in a localized valence space by projection for analysis and transport calculations. We use the cc-pVDZ-DK basis in all calculations.

To get an idea of the largest on-site SOC elements, we calculated the zero-field splitting (ZFS) for the carbon atom. The dominant SOC terms in the Hamiltonian are given by

$$H_{\mathrm{SOC}} = \xi \mathbf{L} \cdot \mathbf{S} \tag{4}$$

in the $2p$ three-orbital subspace. The ^3P ground state of the carbon atom is split into three multiplets ($J = 0$, 1, and 2). The bare matrix element between the atomic $2p$ orbitals is about 5 meV, whereas the ZFS using GHF is about 3 meV. The experimental ZFS is about 2 meV.[10]

For more validation, we also calculated the electronic bands of helicene using an effective tight-binding Hamiltonian,

$$H = -t_i a_{i,\sigma}^\dagger a_{i+1.\sigma} - i\lambda_i a_{i,\sigma}^\dagger a_{i+1.\sigma} + H.c. \tag{5}$$

where t_i and λ_i take one of two values depending on the bond and alternating phase along the chain, respectively. We extracted these parameters by performing an ab initio calculation and projecting to a localized valence orbital space. As analyzed for a Slater-Koster parametrized model of helicene in Geyer et al,[11] the effective SOC terms can arise by combining on-site SOC of carbon with coupling of orbitals on neighboring sites through the nuclear potential and kinetic energy. For helicene, there is an effective SOC generating pathway that is first-order in on-site SOC given by

$$1p_z \xrightarrow{V_{12}} 2p_y \xrightarrow{\text{SOC}} 2p_z \tag{6}$$

where 1 and 2 denote neighboring carbon atoms. Note that there is nonzero coupling V_{12} between $1p_z$ and $2p_y$ orbitals because of the helical curvature and its sign depends on the helicity. The bare magnitude of V_{12} elements is also quite large of the order of 5-10 eV. Therefore, an effective SOC of the order of meV can be generated, consistent with the conclusions of Geyer et al.[11]

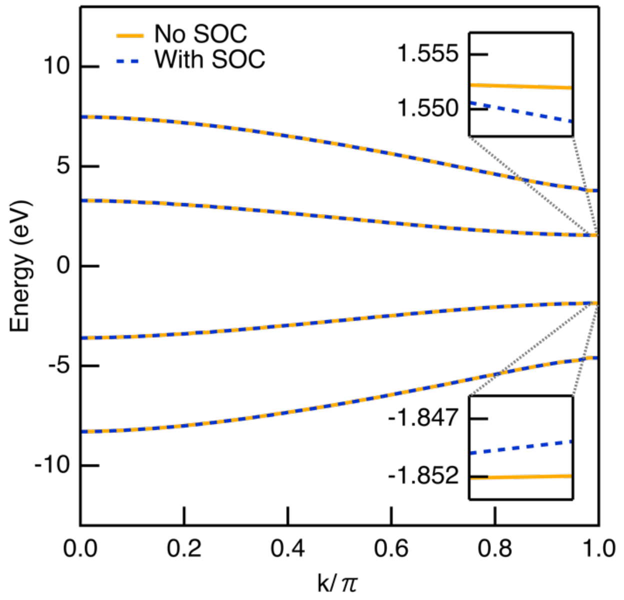

Figure S9. Tight binding bands of Helicene with and without SOC.

Figure S9 shows the calculated bands of helicene with and without SOC following this procedure. In the figure we see four π-band branches – two pairs each corresponding to the inner and outer helices. We see a splitting of the conduction and valence bands of the order of 3 meV at the band edge, negligible compared to the bandwidths which are of the order of eV.

2) Calculation of Transmission and Spin-Polarization Using Green's Function Method

We use non-equilibrium Green's function method to calculate energy-dependent transmission and spin-polarization functions, following the treatment of Tian et al.[12] We obtain the Green's function (G) by calculating the self-consistent solution of

$$G = \left(E - H - \Sigma_l - \Sigma_r - \Sigma_p\right)^{-1} \tag{7}$$

where H is the Hamiltonian obtained from the electronic structure calculation, $\Sigma_{l,r}$ are self-energies due to left/right electrodes, and Σ_p is the self-energy that accounts for electron-phonon coupling, which can be written as

$$\Sigma_p = D_0 G \tag{8}$$

where D_0 represents the electron-phonon coupling strength. The self-energy Σ_p results in further vibronic broadening of the molecular eigenstates, given by $i(\Sigma_p - \Sigma_p^\dagger)$, which is proportional to the spectral function $i(G - G^\dagger)$. This ensures that the broadening at any energy scales with the density of states at that energy. This simple self-energy is an approximation of a more general expression for the phonon self-energy given by

$$\Sigma_p(E) = \frac{i}{2\pi}\int dE' M\, D(E - E')G(E)M \tag{9}$$

where M is the electron-phonon coupling matrix and D is the phonon Green's function. The simpler form emerges when we assume energy-independent coupling from the wide-band limit of gold, and consider only the local density of states contribution. This effectively treats the phonon bath as a source of uniform broadening proportional to the local electronic structure.

The total transmission function $T(E)$ includes both coherent and incoherent processes, and is expressed as[12]

$$T = T_{rl} + \frac{T_{pr}T_{pl}}{T_{pr} + T_{pl}} \tag{10}$$

where the first term represents coherent transmission and the second term accounts for incoherent processes. These transmission components are calculated using

$$T_{\mu\nu} = \mathrm{Tr}\left(\Gamma_\mu G \Gamma_\nu G^\dagger\right) \tag{11}$$

with the broadening functions defined as

$$\Gamma_\mu = i\left(\Sigma_\mu - \Sigma_\mu^\dagger\right) \tag{12}$$

We note that this formalism is accurate only when the vibrational energy is sufficiently small that the density of states can be considered constant over the relevant energy range.

The calculated transmission functions include contributions from four terms, $T_{\uparrow\uparrow}$, $T_{\uparrow\downarrow}$, $T_{\downarrow\uparrow}$, and $T_{\downarrow\downarrow}$, where $T_{\uparrow\uparrow}$, $T_{\downarrow\downarrow}$ represent the spin-up and spin-down transmissions without spin-flipping.

$$P = (T_{\uparrow\uparrow} + T_{\downarrow\uparrow} - T_{\uparrow\downarrow} - T_{\downarrow\downarrow})/T,$$

where T is the total transmission. We note that the molecule is attached to multiple channels in the left and right leads, otherwise the polarization is identically zero in the coherent limit. Figure 5 in the main text shows the coherent contribution to the transmission and spin polarization. The effect of electron-phonon coupling on the spin polarization is shown in Figure S10 for molecule **1**. While there is some enhancement in the polarization with increasing coupling, it remains negligible.

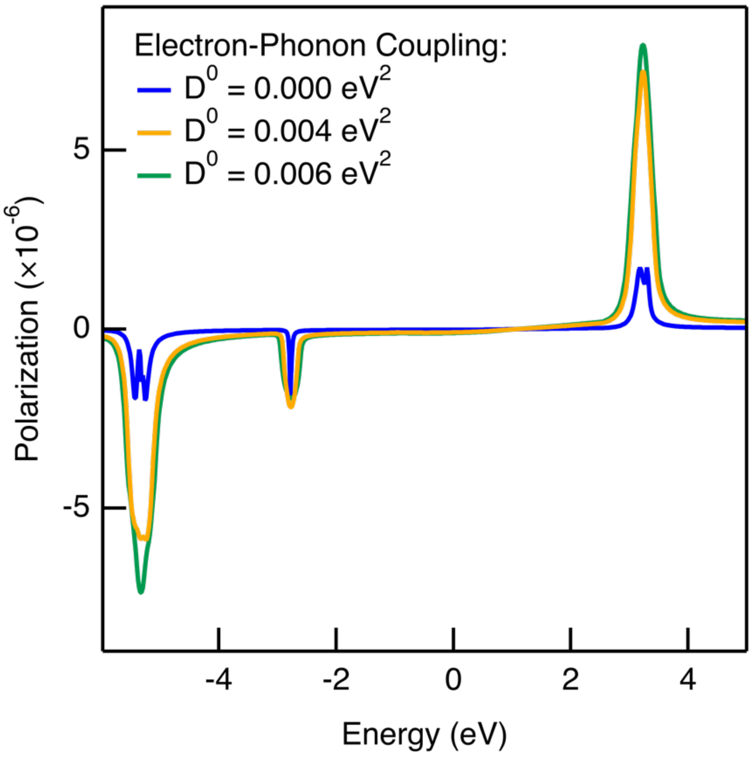

*Figure S10. Spin polarization for molecule **1** for different electron phonon coupling strengths.*

V. References

(1) Lee, W.; Louie, S.; Evans, A. M.; Orchanian, N. M.; Stone, I. B.; Zhang, B.; Wei, Y.; Roy, X.; Nuckolls, C.; Venkataraman, L., Increased Molecular Conductance in Oligo[n]phenylene Wires by Thermally Enhanced Dihedral Planarization, *Nano Lett.* **2022,** *22*, 4919-4924.

(2) Babij, N. R.; McCusker, E. O.; Whiteker, G. T.; Canturk, B.; Choy, N.; Creemer, L. C.; Amicis, C. V. D.; Hewlett, N. M.; Johnson, P. L.; Knobelsdorf, J. A.; Li, F.; Lorsbach, B. A.; Nugent, B. M.; Ryan, S. J.; Smith, M. R.; Yang, Q., NMR Chemical Shifts of Trace Impurities: Industrially Preferred Solvents Used in Process and Green Chemistry, *Organic Process Research & Development* **2016,** *20*, 661-667.

(3) Demuth, J.; Gallego, L.; Kozlikova, M.; Machacek, M.; Kucera, R.; Torres, T.; Martinez-Diaz, M. V.; Novakova, V., Subphthalocyanines as Efficient Photosensitizers with Nanomolar Photodynamic Activity against Cancer Cells, *J. Med. Chem.* **2021,** *64*, 17436-17447.

(4) Lavarda, G.; Labella, J.; Martínez-Díaz, M. V.; Rodríguez-Morgade, M. S.; Osuka, A.; Torres, T., Recent advances in subphthalocyanines and related subporphyrinoids, *Chem. Soc. Rev.* **2022,** *51*, 9482-9619.

(5) Venkataraman, L.; Klare, J. E.; Tam, I. W.; Nuckolls, C.; Hybertsen, M. S.; Steigerwald, M. L., Single-Molecule Circuits with Well-Defined Molecular Conductance, *Nano Lett.* **2006,** *6*, 458-462.

(6) Blum, V.; Gehrke, R.; Hanke, F.; Havu, P.; Havu, V.; Ren, X.; Reuter, K.; Scheffler, M., Ab initio molecular simulations with numeric atom-centered orbitals, *Comp. Phys. Commun.* **2009,** *180*, 2175-2196.

(7) Perdew, J. P.; Burke, K.; Ernzerhof, M., Generalized Gradient Approximation Made Simple, *Phys. Rev. Lett.* **1996,** *77*, 3865-3868.

(8) Lenthe, E. v.; Baerends, E. J.; Snijders, J. G., Relativistic regular two-component Hamiltonians, *J. Chem. Phys.* **1993,** *99*, 4597-4610.

(9) Dyall, K. G., Interfacing relativistic and nonrelativistic methods. I. Normalized elimination of the small component in the modified Dirac equation, *J. Chem. Phys.* **1997,** *106*, 9618-9626.

(10) Ganyushin, D.; Neese, F., First-principles calculations of zero-field splitting parameters, *J. Chem. Phys.* **2006,** *125*, 024103.

(11) Geyer, M.; Gutierrez, R.; Mujica, V.; Cuniberti, G., Chirality-Induced Spin Selectivity in a Coarse-Grained Tight-Binding Model for Helicene, *J. Phys. Chem. C* **2019,** *123*, 27230-27241.

(12) Tian, W.; Datta, S.; Hong, S.; Reifenberger, R.; Henderson, J. I.; Kubiak, C. P., Conductance spectra of molecular wires, *J. Chem. Phys.* **1998,** *109*, 2874-2882.
